# Purtscher-like Retinopathy in a Patient with Systemic Sclerosis: A Case Report and Narrative Review

**DOI:** 10.3390/biomedicines11030839

**Published:** 2023-03-10

**Authors:** Barbara Pieklarz, Ewa Gińdzieńska-Sieśkiewicz, Izabela Zawadzka, Magdalena Bagrowska, Joanna Daniluk, Joanna Konopińska, Otylia Kowal-Bielecka, Diana Anna Dmuchowska

**Affiliations:** 1 Ophthalmology Department, Medical University of Białystok, 24A M. Sklodowskiej-Curie, 15-276 Bialystok, Poland; 2 Department of Rheumatology and Internal Diseases, Medical University of Bialystok, 24A M. Sklodowskiej-Curie, 15-276 Bialystok, Poland

**Keywords:** Purtscher-like retinopathy, systemic sclerosis, scleroderma, occlusive microangiopathy, optical coherence tomography

## Abstract

Purtscher-like retinopathy (PLR) is an uncommon occlusive microangiopathy associated with various systemic conditions. We report a case of PLR related to severe progressive systemic sclerosis (SSc), an autoimmune disease characterized by widespread angiopathy and fibrosis, in a 44-year-old Caucasian male diagnosed with early diffuse cutaneous systemic sclerosis (dSSc). Upon ophthalmological examination, pathognomonic fundoscopy abnormalities were found. Spectral domain optical coherence tomography (SD-OCT), angio-OCT, and visual field results are documented at initial diagnosis and follow-up visits. The detailed ophthalmological assessment is juxtaposed with rheumatological evaluation and treatment. Current literature on probable pathophysiological mechanisms is reviewed in accordance with the described case. The PLR seems to be connected to severe SSc-related angiopathy initiated by capillary endothelial damage, with ultimate arteriolar precapillary occlusion in the inner retinal layer. Although this is not routinely recommended, we suggest that ophthalmological examinations may be advantageous in patients with SSc, as serious eye pathology may be present despite the lack of symptoms reported by the patient. Patients with PLR require a differential diagnosis and regular follow-up. Proper treatment of the underlying disease may have beneficial effects on the natural course of PLR.

## 1. Introduction

Purtscher-like retinopathy (PLR) is an uncommon occlusive microangiopathy associated with various systemic conditions. The most common causes are acute pancreatitis, renal failure, and autoimmune diseases, including systemic lupus erythematosus (SLE) and dermatomyositis [1,2,3,4]. The pathogenesis remains unclear, and several mechanisms have been proposed to explain the characteristic fundus findings. The most likely theory is precapillary occlusion due to fat emboli, pancreatic protease, or activation of complement factors resulting in leukocyte, fibrin, and platelet aggregation involving both retinal and choroidal vessels. Other mechanisms have also been discussed [5,6]. We report a case of PLR related to severe progressive systemic sclerosis (SSc), an autoimmune disease characterized by widespread angiopathy and fibrosis. To the best of our knowledge, this is the third reported case of the coexistence of these entities in the literature; however, in previous SSc cases, simultaneous renal crisis was also diagnosed, which was not the case in the described patient [7,8].

## 2. Case Report

**Figure 1:**
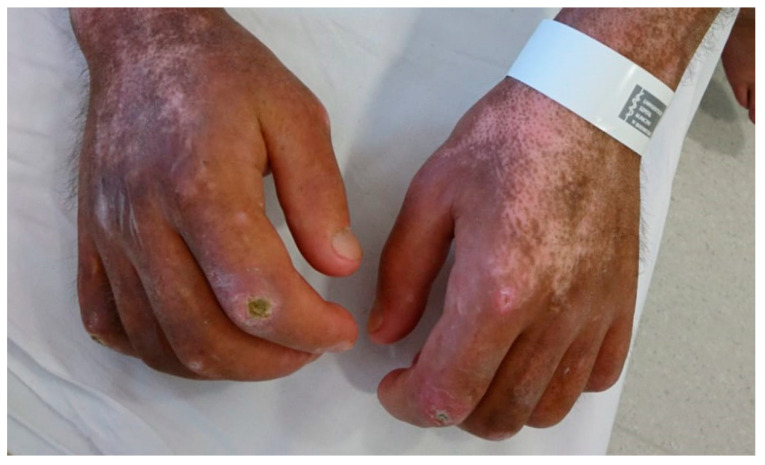
Painful ulcers and skin thickening of both hands.

**Figure 2:**
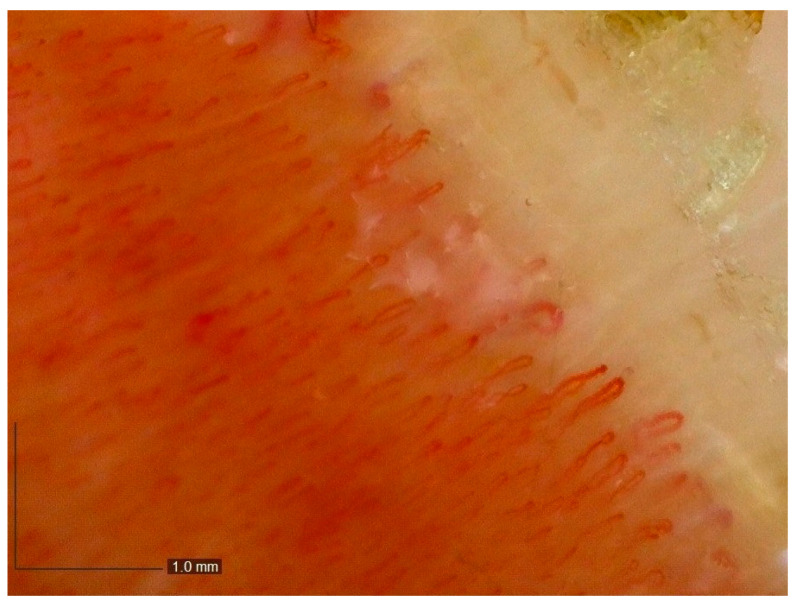
Nailfold capillaroscopy demonstrating severe microangiopathy.

A 44-year-old Caucasian male diagnosed with early diffuse cutaneous systemic sclerosis (dSSc) (anti-Scl-70 antibodies positive (+++)) with a duration of 3 months was admitted to the Department of Rheumatology, Medical University of Bialystok, Poland, due to progressive skin thickening, painful ulcers on both hands (Figure 1), and signs of heart involvement (high troponin and N-terminal pro b-type natriuretic peptide (NT-proBNP) concentrations). No significant changes in the coronary arteries were found on coronarography. The results of the laboratory tests are presented in Table 1. The patient’s medical history included hypertension, dyslipidemia, and alcohol dependence syndrome. During hospitalization, heart involvement due to dSSc was confirmed by MRI, while nailfold capillaroscopy revealed severe microangiopathy typical of dSSc (Figure 2).

**Figure 3:**
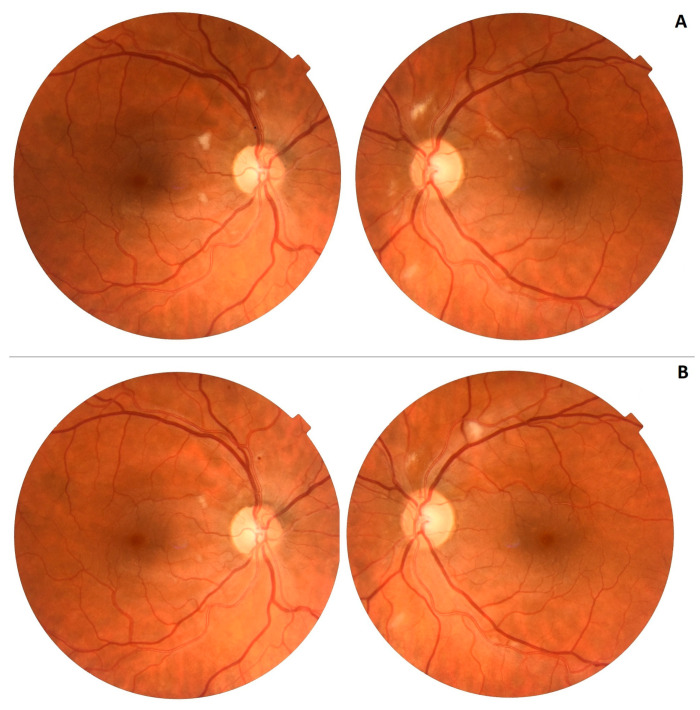
(**A**). Fundoscopy of RE and LE at diagnosis: cotton-wool spots and Purtscher flecken. (**B**). Fundoscopy of RE and LE showing reduced cotton-wool spots at 1 month follow-up.

The patient had no previous history of severe trauma or ocular disease, and he denied any visual disturbance. Upon routine ophthalmological examination, the patient presented full uncorrected visual acuity (VA) bilaterally. The intraocular pressure (IOP) in the right eye (RE) and left eye (LE) was 17 and 16 mmHg, respectively. Slit-lamp examination revealed lid stiffness and telangiectasia on the upper and lower lids of both eyes, without any abnormalities in the anterior segment. Fundoscopy revealed cotton-wool spots confined to the posterior pole surrounding the optic disc and subtle Purtscher flecken, discrete areas of retinal whitening between the arterioles and venules (pathognomonic sign), in both eyes (Figure 3A).

**Figure 4:**
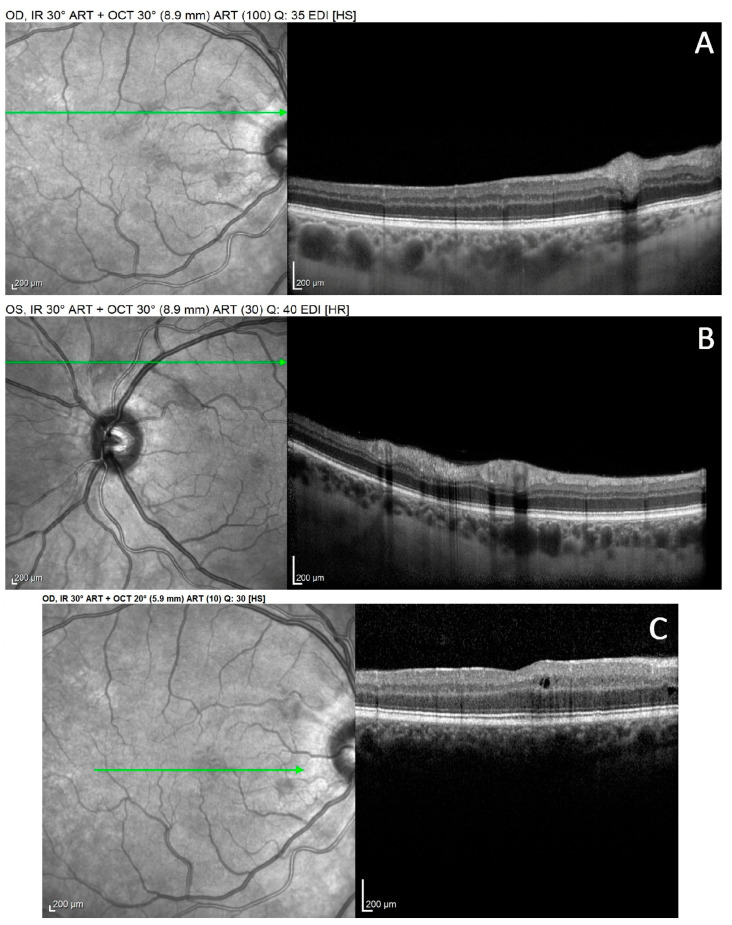
SD-OCT: hyperreflectivity in retinal nerve fiber layer corresponding to cotton-wool spots in (**A**) RE and (**B**) LE; (**C**) macular cystoid spaces in RE.

**Figure 5:**
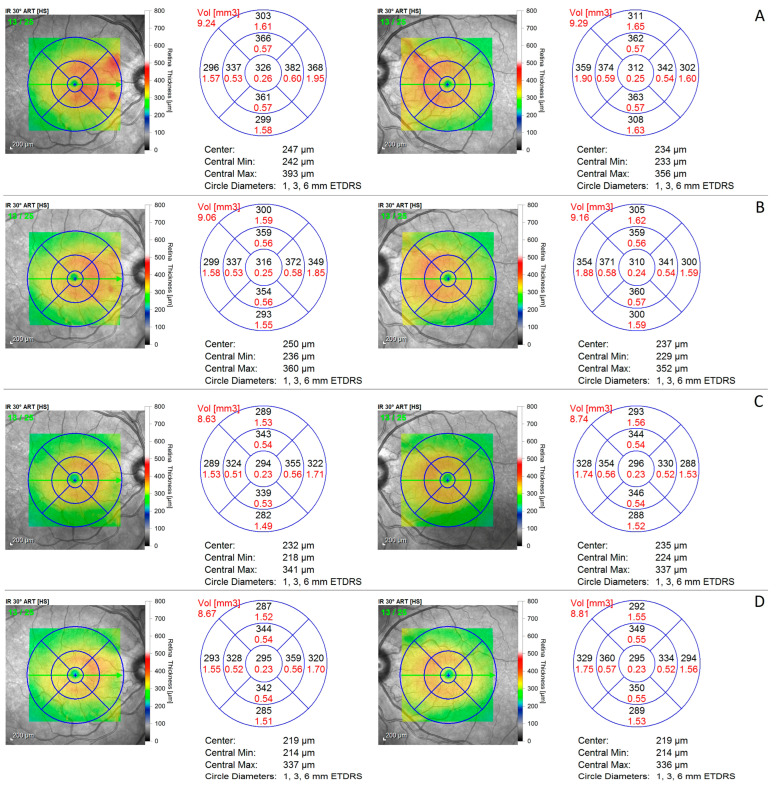
(**A**). Initial OCT thickness mapping of RE and LE. (**B**). OCT thickness mapping of RE and LE 4 weeks later, showing reduction. (**C**). OCT thickness mapping of RE and LE 6 months later showing further reduction. (**D**). OCT thickness mapping of RE and LE around 12 month follow-up, showing stabilization.

Spectral-domain optical coherence tomography (SD-OCT; Heidelberg Engineering, Heidelberg, Germany) of both eyes showed hyperreflectivity in the nerve fiber layer, corresponding to cotton-wool spots and slight cystoid spaces in the macula of the RE (Figure 4). OCT thickness mapping of both eyes revealed retinal thickening, particularly in the papillomacular bundle (Figure 5A).

**Figure 6:**
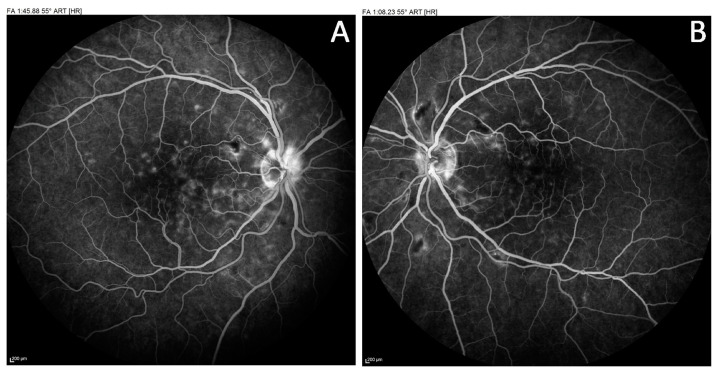
Fluorescein angiography of (**A**) RE and (**B**) LE reflecting vascular wall enhancement, vein dilatation, and capillary occlusion, as well as slight leakage within macula and optic disc in RE.

**Figure 7:**
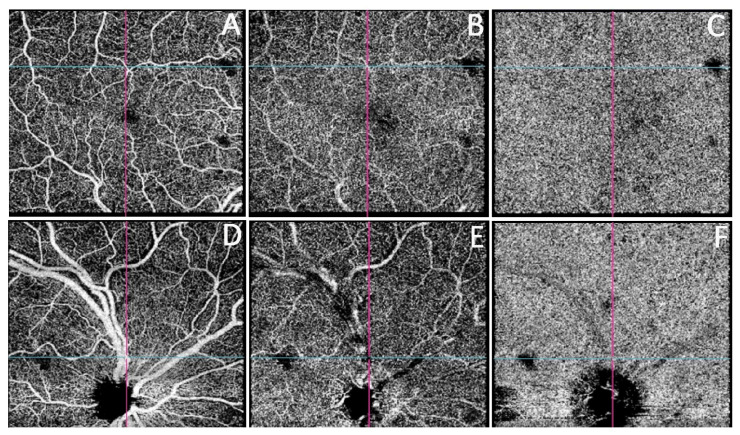
OCT-A of RE reflecting local deficiency within superficial and deep capillary plexus and choriocapillaris. (**A**–**C**) Macular area and (**D**–**F**) peripapillary area: (**A**,**D**) superficial plexus, (**B**,**E**) deep plexus, (**C**,**F**) choriocapillaris.

Fluorescein angiography of both eyes revealed vascular wall enhancement, vein dilatation, and capillary occlusion corresponding to the cotton-wool spots, as well as slight leakage within the macula and optic disc in the RE (Figure 6). OCT angiography (OCTA; Canon, Tokyo, Japan) of both eyes showed superficial and deep capillary occlusion within the areas of cotton-wool spots and the region of macular edema (in RE). A hypointense signal pattern in the choriocapillaris layer was also observed (Figure 7).

**Figure 8:**
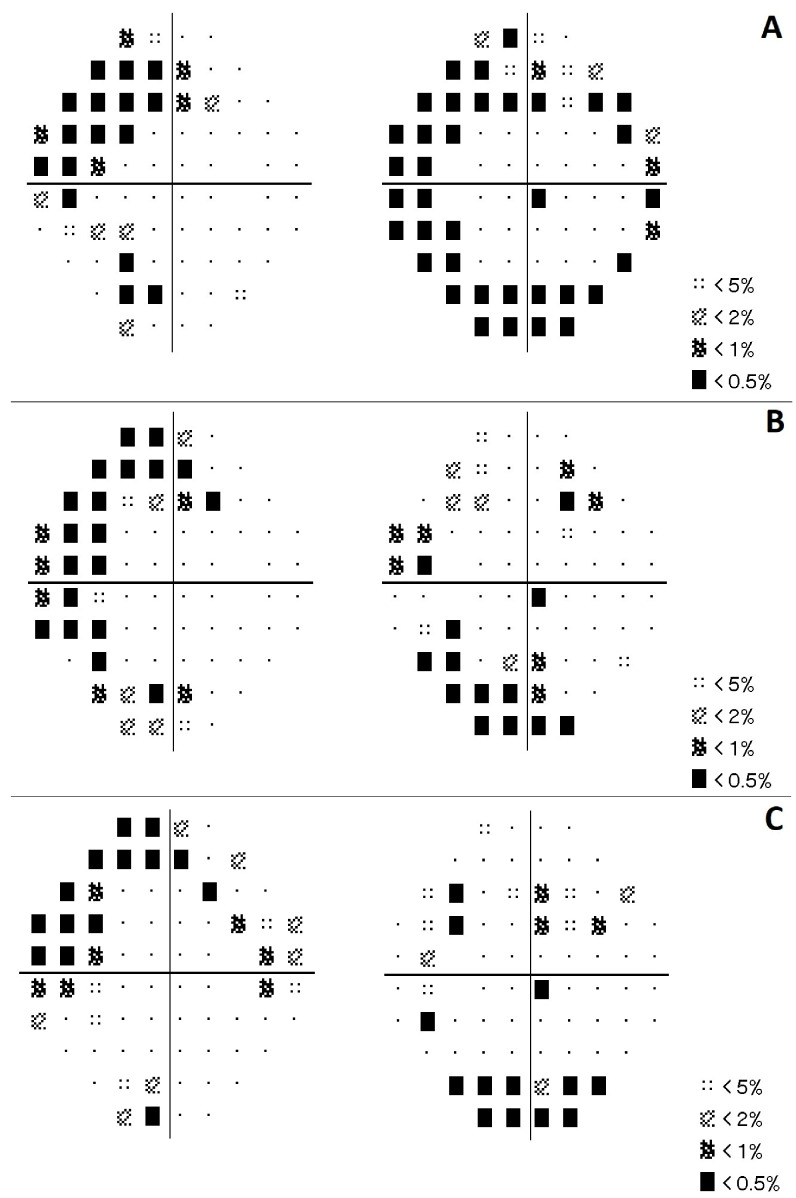
(**A**). Visual field test (30-2) of RE and LE. (**B**). Visual field test (30-2) of RE and LE 4 weeks later. (**C**). Visual field test (30-2) of RE and LE at 12 month follow-up.

A visual field test using a Humphrey visual field analyzer (Carl Zeiss AG, Jena, Germany) with the 30-2 SITA Standard algorithm revealed arcuate scotoma in the RE and diffuse double arcuate scotoma in the LE (Figure 8A). A 120-point visual field test confirmed mid-peripheral visual dysfunction. Because of the severe course and rapid progression of dSSc with heart involvement, the patient received intravenous tocilizumab (560 mg) and corticosteroids. Moreover, methotrexate therapy was intensified from 10 mg to 15 mg per week, and vasodilatory treatment was initiated. Follow-up evaluation 1 month later revealed improvement of skin changes and healing of the ulcers. Fundoscopy showed reduced cotton-wool-spot density in both eyes (Figure 3B) and reduced macular thickness of the retina in both eyes, with resolution of cystoid spaces in the RE on SD-OCT (Figure 5B). Visual field defects changed in the RE (mean defect (MD) from −5.84 to −6.06) and LE (MD from −10.89 to −4.08) (Figure 8B). At 6 and 12 month follow-up, no cotton-wool spots were observed on the fundus of either eye, and the thickness of the macular retina had stabilized (Figure 5C,D). Visual field defects in the LE at 12 month follow-up showed partial improvement (MD = −4.95) and persistent defects in the RE (MD = −5.19) (Figure 8C).

Over a period of almost 12 months, the patient received nine infusions of tocilizumab, and methotrexate treatment was intensified (20 mg per week). Laboratory tests showed normalization of the troponin and NT-proBNP concentrations, with a tendency to decrease compared to previous hospitalizations (Table 1).

## 3. Discussion

Purtscher’s retinopathy (PUR) occurs primarily as a result of cranial trauma or thoracic compression. However, in cases of nontraumatic etiology, the correct term is Purtscher-like retinopathy (PLR) [5]. The most common causes of PLR are acute pancreatitis, renal failure, and autoimmune diseases. Other possible causes of PLR include various conditions and diseases, such as hemolytic uremic syndrome (HUS) [9] and hemolysis, elevated liver enzymes, and low platelets (HELLP) syndrome [10]. Other, rarer causes of PLR include pre-eclampsia, [11] neoplastic diseases [12,13], and viral infections such as COVID-19 [14], influenza [15], or dengue [16]. PLR following childbirth [17] or as a rare complication of retrobulbar anesthesia [18] or a result of the Valsalva maneuver [19] has also been reported in the literature. On the other hand, other diseases can sometimes present with similar fundus findings, such as cytomegalovirus retinitis, HIV, leukemia, diabetes, and hypertensive retinopathy [20]. In the presented case, a detailed differential diagnosis was performed. The laboratory tests, including HIV, COVID-19, and CMV infection, tumor markers, blood morphology with smear, glucose levels, and hepatic parameters, as well as imaging tests (abdominal computed tomography scan) did not reveal any possible causes of PLR other than SSc in our patient. Moreover, blood pressure remained within the normal range.

The pathogenesis of PUR/PLR remains unclear, and several mechanisms have been proposed to date. Arteriolar precapillary occlusion resulting in an infarct of the retinal nerve fiber layer appears to be the most accepted and consistent possibility. Fat emboli, pancreatic protease in systemic circulation, or leukocyte aggregation and C5 complement activation can lead to microembolization. Other, rarely proposed pathogenic processes include vascular endothelial dysregulation and capillary endothelial damage followed by endothelin-induced vasculopathy [21]. Harrison et al. noted that in contrast to the choriocapillaris, the inner retinal microvascular system is uniquely vulnerable to endothelial dysregulation [21]. Retinal circulation is characterized by a low flow with high-level oxygen extraction and is mainly determined by autoregulatory mechanisms and local factors, while the choroidal blood flow is mainly controlled by autonomic innervation [22]. Endothelial dysregulation is seen in various diseases in which vasoconstrictive substances are overproduced, and a number of conditions are associated with systematically elevated endothelin-1, such as renal failure, hemolysis, HELLP syndrome, and Still’s disease. Cases of PLR presenting with these conditions have been reported in the literature [21]. In our study, we were able to visualize microvascular damage using fluorescein angiography and OCT-A. SD-OCT demonstrated corresponding edema within the retinal nerve fiber layer. The visual field reflected the functional consequences.

Choroidal vessel involvement in PUR/PRL has also been discussed. In one study, indocyanine green angiography revealed areas of choroidal hypofluorescence, and the authors suggested that the involvement of the choroidal vasculature may occur more often than expected and may be associated with a worse prognosis for vision improvement [23]. Histopathology and electroretinography results and late changes at the RPE level visualized upon SD-OCT and described in the literature provide further evidence of choroidal involvement in PUR/PRL [6]. Other studies have suggested that a honeycomb-like hypointense signal from the choriocapillaris layer on OCT-A indicates the involvement of the choroid with ischemia [24]. A hypointense signal pattern in the choriocapillaris layer observed on OCT-A was also found with our patient, most likely as an artifact projection from the retinal layers.

The most frequently reported ocular manifestation of SSc is keratoconjunctivitis sicca, but skin changes of the eyelids (such as lid stiffness and tightness) are the most consistent features related to the underlying disease in patients with SSc [25]. Kreps et al. emphasized that more studies are needed to elucidate the extent of ocular involvement in SSc and its correlation with disease progression [25]. Endothelial damage followed by an autoimmune response and inflammation resulting in diffuse fibrosis is the underlying cause of SSc. Microvascular damage occurs due to endothelial dysregulation, as manifested by increased production of vasoconstrictors (vascular endothelial growth factor A (VEGF-A) and endothelin-1 (ET-1)), underproduction of vasodilators (NO and prostacyclin), and, finally, a defective response of damaged endothelial cells. Overproduction of ET-1 in the skin, lung tissue, and serum of SSc patients leads to organ complications [26]; these are also involved in autoregulation of the retinal microvascular system. Therefore, in our patient, PLR seemed to be connected to severe SSc-related angiopathy. The aggravators in the two previously described cases of PLR in patients with systemic scleroderma were dual-vessel microangiopathy and scleroderma renal crisis, which involve the development of thrombotic microangiopathy followed by accelerated-phase hypertension and, finally, progressive acute kidney injury. In both cases, the VA was reduced [7,8]. Kidney function in our patient remained normal. Additionally, elevated values of laboratory indicators of heart injury were observed, without significant changes in the coronary arteries. Direct heart injury (heart fibrosis) was confirmed by MRI. The presence of cardiac involvement is generally associated with poor prognosis in patients with SSc [27]. One case of PLR associated with myocardial infarction has been reported in the literature, but this patient underwent emergent angioplasty of his coronary vessels, and also experienced a transient ischemic attack (TIA) [28].

It is worth noting that among autoimmune diseases, PLR is most frequently described as coexisting with SLE; however, SLE is more common than SSc. It has been reported that PLR is a devastating and severe complication of SLE, and an association of PLR with central nervous system involvement in lupus and highly active disease has been observed. It has been hypothesized that endothelial damage related to vasculitis could play a key role in the pathogenesis of PLR in patients with SLE [3].

Most cases of PLR are bilateral, with variability in visual disturbances (asymptomatic full VA to light perception) [5]. The most frequently reported signs of PLR are cotton-wool spots (as a sign of an infarct of the retinal nerve fiber layer), hemorrhages, and Purtscher flecken (as a result of occlusion of the precapillary arterioles). Less frequently, macular edema, optic disc swelling, or a pseudo-cherry-red spot can also occur [5]. Macular edema with the presence of serous subretinal fluid has also been reported in the literature [4]. In our case report, we observed cotton-wool spots, Purtscher flecken, and macular edema without subretinal fluid.

Regular follow-up of patients with PLR is worth consideration. Harrison et al. suggested examination by an ophthalmologist with funduscopic evaluation at least 1 month, 2 months, and 6 months after initial diagnosis. Although normalization of the retinal appearance has been observed in 40% of patients, optic nerve atrophy, mottling of the retinal pigment epithelium, retinal thinning, and narrowing of the retinal arteries can occur [5]. Recurrence of PLR and progression to neovascularization and vitreous hemorrhage have also been reported in the literature, and it has been emphasized that the course of PLR may be chronic and progressive [29]. The possibility of serious complications confirms the need for regular monitoring of PLR patients.

Our case report has some strengths, including a relatively long follow-up period and detailed imaging results change during this period. The study has some potential limitation. As our patient did not report any visual disturbances (despite the detection of a significant visual field defect), we do not know at what stage of the disease the PLR diagnosis was made. It is possible that fundus changes might have been more advanced. Due to the rarity of the disease this is a case report and not a case series.

At present, there are no clear treatment recommendations for PLR due to the rarity, varied etiology, and heterogenous course of this condition. Further studies are needed to determine whether treatment could change the natural course of the condition. The effectiveness of corticosteroid treatment has not been proven [5]. Proper treatment and control of underlying disease is crucial; from an ophthalmological point of view, this proved beneficial in our case. On the other hand, ophthalmic treatment certainly depends on the advancement of ischemic changes and their possible complications. This was not indicated in the described case. The patient remains under regular follow-up care. According to the patient’s report, the subjective daily functioning associated with the underlying disease has improved owing to effective treatment of systemic sclerosis.

## 4. Conclusions

In our patient, PLR seemed to be connected to severe SSc-related angiopathy initiated by capillary endothelial damage, with ultimate arteriolar precapillary occlusion in the inner retinal layer.

Our case shows that although it is not routinely recommended, ophthalmological examination may be advantageous for patients with SSc, as serious eye pathology may be present despite a lack of symptoms reported by the patient. Patients with PLR require a differential diagnosis and regular follow-up. Proper treatment of the underlying disease can have a beneficial effect on the natural course of PLR.

## Figures and Tables

**Table 1:** Results of laboratory blood tests at initial visit and 6 and 12 month follow-up.

Parameter, Unit	Normal Range	At Initial	6-Month Follow-Up	12-Month Follow-Up
High-sensitivity troponine [ng/L]	up to 32	911.80	190	31.40
NT-proBNP [pg/mL]	0.0–125.0	4344	1765	1289
Creatine Kinase-MB (activity) [IU/L]	0.0–25.0	188	35	27
CRP [mg/L]	0.0–10.0	34.5	<1	11.1
ESR [mm/1 h]	up to 8	52	1	19
ESR [mm/2 h]	up to 8	84	2	39
Creatinine [mg/dL]	0.73–1.18	0.70	0.67	0.68
Uric Acid [mg/dL]	3.5–7.2	6.7	8.8	4.5
IL-6 [pg/mL]	1.5–7.0	296.5	148.4	18.3

Abbreviations: NT-pro-BNP, N-terminal pro b-type natriuretic peptide; CRP, C-reactive protein; ESR, erythrocyte sedimentation rate; h, hours; IL-6, interleukin 6.

## Data Availability

All the materials and information will be available upon an e-mail request to the corresponding author. Name and exact data of the participant of the study may not be available owing to patient confidentiality and privacy policy.
